# Does governance impact on the financial development-carbon dioxide emissions nexus in G20 countries

**DOI:** 10.1371/journal.pone.0273546

**Published:** 2022-08-22

**Authors:** Ya Wen, Pingting Song, Deyong Yang, Chen Gao

**Affiliations:** 1 School of Economics, Beijing Technology and Business University, Beijing, China; 2 Accounting School, Harbin University of Commerce, Harbin, China; 3 China Economics and Management Academy, Central University of Finance and Economics, Beijing, China; University of Pisa, ITALY

## Abstract

In the past 40 years, the continuous strengthening of the greenhouse effect has led to a significant increase in the global average temperature. Although people’s understanding of climate change has been strengthened, the world has not yet witnessed a significant decline in pollutant emissions; hence it is imperative to get to the root cause. This paper is based on the STIRPAT model framework and uses the panel data of G20 countries over the period 1999–2019 to examine the role of financial development on carbon emissions under good governance. The results show that financial development significantly promotes carbon dioxide emissions, and the impact presents an inverted “U”-shaped trend when the quadratic term of financial development is introduced. Surprisingly, governance quality indicators increase carbon emissions. However, financial development accompanied by good governance suppresses carbon emissions. Moreover, according to the grouped results of developed and developing countries, different nations should adopt differentiated strategies in development finance to implement the carbon emission targets proposed by the G20. In addition, this paper also confirms the existence of the Environmental Kuznets Curve hypothesis. In light of this, policymakers should optimize the quality of governance while shifting their agendas toward environmentally responsible financial practices to promote financial development to improve environmental quality effectively. Furthermore, strengthen international cooperation, enhance public environmental protection concepts, and take joint actions to achieve low-carbon and win-win results.

## 1. Introduction

In recent years, environmental degradation and climate change have been the most thorny issues for policymakers of nations around the globe [1]. As carbon dioxide in greenhouse gas emissions as a share must, therefore, become one of the hotspots in climate change research, many countries are dedicated to developing appropriate energy policies [2]. In this context, the G20 leaders reaffirmed their commitment to fully and effectively implementing the United Nations Framework Convention on Climate Change and the Paris Agreement. However, the report issued by the Intergovernmental Panel on Climate Change (IPCC) pointed out that the global temperature has risen by 1.5°C on average, indicating that the role of energy conservation and emission reduction is already quite severe and urgent in the future.

**Fig 1:**
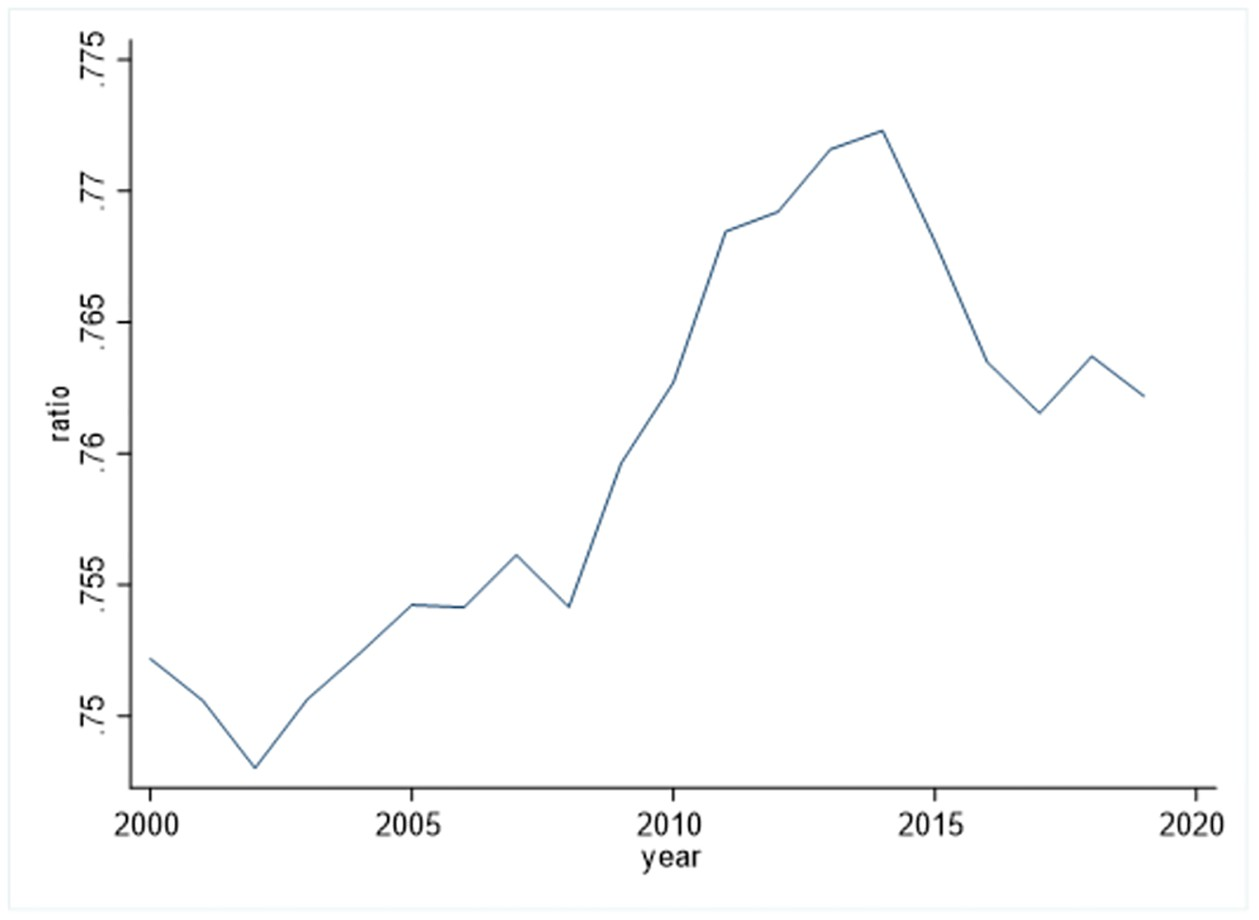
The ratio of G20 countries’ total CO2 emissions to the world’s.

Most notably, the G20 group includes the world’s most developed countries and emerging market economies. Fig 1 displays the ratio of G20 to the world in the case of carbon emissions. We can find that from 2002 to 2014, this ratio rose in fluctuations and reached a peak value of 0.772 in 2014, reflecting that the total CO2 emissions of G20 countries accounted for a large proportion of the world, and they are the main CO2 emitters over the world. In terms of greenhouse gas emission reduction, they bear the inescapable international responsibility, and their practical effect will affect the trend of global warming [3]. Research on these countries is therefore crucial, and that is why we chose the G20 nations as our research sample.

Existing literature on the nexus between environmental degradation and economic development, known as the Environmental Kuznets Curve (EKC), is supported by research in different regions [4, 5]. Undeniably, financial development has the key active element of carbon emissions, while the results of the existing literature on its relationship are at odds with one another [6, 7]. Therefore, the actual relationship still requires specific and in-depth exploration.

The aggravation of environmental degradation is a major challenge to the nation’s governance capacity [8]. Actively responding to global climate change and making substantial reforms regarding energy structure and carbon emissions are in line with several sustainable development goals of the United Nations. Besides, good governance has been paid more and more attention by researchers, with the aim of curbing the decline of environmental quality [9]. Nonetheless, to the best of our knowledge, the existing literature on the role of governance in environmental degradation is still relatively limited. Accordingly, this paper attempts to examine the effect of governance quality and financial development in G20 countries on carbon emissions.

Contributions of this paper: The current research is unique as it contributes to the stock of literature in many ways. First: This paper introduces governance into the relationship between financial development and carbon emissions in G20 countries to investigate whether and how governance functions as a moderator. Second: Few researchers have evaluated the impact of governance quality on CO2 emissions and how governance affects the financial sector to improve environmental quality. Moreover, no article has studied the combined effect of the two on the level of the environment of G20 nations. Third: Considering the conspicuous differences between developed and developing countries in governance, financial levels and carbon emissions, we divide the sample into two groups according to the degree of development and re-estimate to eliminate heterogeneity. Fourth: We consider two categories of financial development (financial institution development and financial market development) and three dimensions of governance (institutional governance, economic governance and political governance) to conduct further robust analysis. The division of categories and the introduction of interaction terms allow us to understand which categories of indicators are the best complementary combinations.

The rest of this article is outlined below: Section 2 presents the review of the literature, followed by Section 3, which introduces data and methodology. Section 4 displays the empirical results and discussion, and Section 5 concludes the study and proposes relevant recommendations.

## 2. Literature review

### 2.1. Financial development and CO2 emissions

Burgeoning literature related to the ecological economy has confirmed that financial development has a positive spillover effect on carbon emissions [10, 11]. The main reason can be considered as a well-functioning financial sector may provide more financing at a lower cost, thus more and more capital flows into environmental protection and energy conservation projects and enterprises, promoting the rapid development of the environmental protection industry and driving the green transformation and upgrading of traditional industries as well [10]. Besides, the development of finance prompts listed companies to adopt energy-saving technologies and finally achieve the goal of reducing environmental pollution [12, 13]. Likewise, Shahbaz et al. [14] showed that inefficient use of energy has a significant and negative influence on environmental quality, while the adoption of high-efficiency technologies at the production and consumption levels can build a cleaner environment. In another study, Shahbaz et al. [15] investigated the nexus between economic growth, energy consumption, financial development, trade openness and CO2 emissions in Indonesia. They concluded that there is a long-term cointegration relationship between variables, and financial development is negatively correlated with CO2 emissions.

On the contrary, the role of financial development could not essentially be anticipated to be negative all the time [16]. For instance, Shahbaz et al. [17] applied the ARDL boundary test method to co-integration in a time-series study to test the association between financial development and CO2 emissions in Malaysia. Their findings suggest that developed financial markets attract more investment, which accelerates the process of industrialization. But it also increases energy demand, which ultimately leads to higher levels of CO2 emissions. Similarly, Bekhet et al. [18] explored the link between CO2 emissions, financial development and energy consumption in the GCC countries by establishing dynamic simultaneous equation models and ARDL boundary tests. Their analysis showed that financial development exacerbates the deterioration of environmental quality in Saudi Arabia, Oman, Qatar and Kuwait. Moreover, consumers can easily obtain consumer loans from financial intermediaries to purchase more household items such as washing machines, air conditioners, and cars, directly or indirectly increasing CO2 emissions [19]. In addition, Muhammad et al. [20] stated that international trade contributes to CO2 emissions, and an inverted “U” relationship exists between urbanization and CO2 emissions.

In the existing literature, studies have also discovered that financial development has an insignificant impact on carbon emissions [21]. Ozturk and Acaravci [22] empirically explored the long-term and causal relationship between energy consumption, economic growth, openness and financial development in Turkey and documented that financial development had no effect on per capita CO2 emissions. Cowan et al. [23] found no correlation between electricity consumption and economic growth in Brazil, India and China in a study of BRICS countries. Based on what has been stated so far, we can conclude that the impact of financial development on carbon emissions is not a simple linear relationship, and the differences in empirical results may be derived from data sources, sample intervals, selected proxy variables and control variables.

### 2.2. Governance quality and CO2 emissions

The previous empirical literature has identified good governance as an important factor in the SDGs [24]. In a recent study, Güney [25] used data from 2005–2018 for 35 countries at different income levels to explore the impact of solar energy and governance on carbon emissions, and denoted that good governance and investment in solar equipment are critical to reducing carbon emissions. Likewise, Baloch and Wang [26] incorporated the governance of carbon emissions into the framework of the EKC to analyse the governance behaviour of CO2 emissions in BRICS countries and implied that governance has a considerable negative correlation with carbon emissions.

Furthermore, the exploration of political corruption in developing countries has attracted attention from researchers. Generally speaking, worsening corruption could make government departments more vulnerable to capture by capital, making governance deviate from sustainable development direction [27]. For a panel of 99 developing countries in multiple regions across the globe, the sub-indicators of governance: political stability, the rule of law and corruption control have been found to be significantly negatively related to per capita CO2 emissions [28]. According to [29], corruption in the government sector in developing countries should be responsible for environmental pollution. They also believe that increasing transparency and improving governance quality can be attached to the confrontation with the challenges of reducing carbon emissions. In addition, Asongu and Odhiambo [30] found that regulatory quality and institutional governance negatively affected inclusive development by regulating CO2 emissions, whereas the interaction term was positively related to inclusiveness, suggesting that better governance is required to achieve sustainable development goals.

### 2.3. Governance, financial development and CO2 emissions

Regarding the nexus between governance and financial development, existing research supports that good governance plays a positive role in the development of finance [31]. For instance, Sarhangi et al. [32] analysed the effect of effective governance and regulatory quality on financial development in Iran by referring to STAR, and they argued that governance quality brings new potentials for financial development. At the same time, good governance can also boost private investment [33], FDI [34], and firm financial performance [35]. Concerning the research on the moderating role of governance or institutions between economic development and carbon emissions, Tamazian and Rao [36] confirmed that governance accommodates the nexus of financial development-environmental degradation. They also held the view that a high level of environmental quality is a positive outcome of the good situation of strong governance, but without perfect institutional arrangements, financial development cannot effectively achieve carbon emission reduction. For a panel of 85 developed and developing economies over 22 years, Bhattacharya et al. [37] claimed that institutional quality and renewable energy contribute to economic output, but are negatively correlated with carbon emissions. Moreover, Wawrzyniak and Doryń [38] took government efficiency and corruption control as an indicator of institutional quality and advocated that as institutional quality improves, it may shrink the growth of CO2 emissions. Furthermore, Farooq [39] employed the dynamic impact of governance on the link between FDI, foreign aid and carbon emissions in Asian economies and identified that better governance could limit pollution from the industrial sector, as well as hinder carbon emissions from inflows of FDI.

## 3. Data and methodology

### 3.1. Variables and data description

Using the panel data of G20 countries over the period 1999–2019, this paper examines how the quality of governance affects the role of financial development in reducing carbon emissions. The availability of statistics on governance quality indicators determines the choice of data starting period. The data is obtained from the International Energy Agency (IEA), the International Monetary Fund (IMF), the World Governance Indicators (WGI), and the World Development Indicators (WDI). Table 1 summarizes the description and sources of each variable.

**Table 1:** Definition and source of the used data.

Variables	Definition	Source
CO2 emissions (*CO*)	CO2 emissions per capita	IEA
Financial development (*FD*)	Financial development index	IMF
Financial institutions development (*FI*)	Financial institutions development index	IMF
Financial markets development (*FM*)	Financial markets development index	IMF
Governance quality (*WGI*)	Global governance indicators	WGI
Political governance (*POL*)	(Political stability+voice and accountability)/2	WGI
Economic governance (*ECO*)	(Regulatory quality+government efficiency)/2	WGI
Institutional governance (*INS*)	(The level of rule of law+corruption control)/2	WGI
Economic development (*RGDP*)	Real per capita GDP	WDI
Trade openness (*TRA*)	The value of trade in goods and services as a share of GDP	WDI
Population size (*POP*)	The total population	WDI
Urbanization (*URB*)	The urban population as a share of the total population	WDI
Industrialization (*IND*)	The industrial value added as a share of GDP	WDI

CO2 emissions (*CO*): the dependent variable. Referring to [40], we apply per capita CO2 emissions in metric tons as a measure of environmental degradation, with data from the IEA database.

As we have stated in the last section, two independent variables are analysed as the determinants of carbon emissions.

Financial development (*FD*): As the modern financial system becomes more diverse, using a single index as an indicator of financial development is flawed. In this sense, the IMF constructs a financial development index based on financial institutions and financial markets regarding depth, access and efficiency. This paper uses the index as a proxy variable for financial development, and the data is collected from IMF databases.

Governance quality (*WGI*): We introduce governance quality as a policy variable into the model to investigate the impact of financial development on carbon emissions in its context. Six dimensions of governance quality are included, namely, political stability (*PS*), voice and accountability (*VA*), regulatory quality (*RQ*), government efficiency (*GE*), corruption control (*CC*) and the level of the rule of law (*RL*). Referring to [41]’s classification of governance quality, they are divided into the following three categories: political governance (*PS*&*VA*), economic governance (*RQ*&*GE*) and institutional governance (*CC*&*RL*). The data for these indicators comes from the WGI database.

Apart from the two independent variables, we also propose other determinants of CO2 emissions, including the level of economic development (*RGDP*), which is measured by real per capita GDP applying the 1999 base period. The squared term of per capita GDP (*RGDP*^*2*^) is incorporated into the model to test the validity of the EKC hypothesis. The level of trade openness (*TRA*) is expressed in the value of trade in goods and services as a share of GDP. The population size (*POP*) is defined as the total population. The urbanization level (*URB*) is measured by the urban population as a percentage of the total population. The level of industrialization (*IND*) is expressed in industrial value added as a share of GDP.

### 3.2. Econometric model and estimation procedures

Financial development not only optimizes the industrial structure and encourages sustainable economic development, but also affects carbon emissions in the process. Based on the IPAT model proposed by [42], the STIRPAT model is widely regarded as the basic framework for examining the role of economic activities on environmental pollution [43]. The basic form of the model is as follows:

$$I_{t}=\alpha_{t}P_{it}^{b}A_{it}^{C}T_{it}^{d}e_{it}$$ (1)

where *I* represents environmental pollution, *P* is the population size, *A* indicates the Economic prosperity, *T* is the technology and *e* devotes the error term. We take the natural log of (1) into the following form:

$$lnI_{t}=\alpha_{t}+blnP_{it}+clnA_{it}+dlnT_{it}+e_{it}$$ (2)

**Fig 2:**
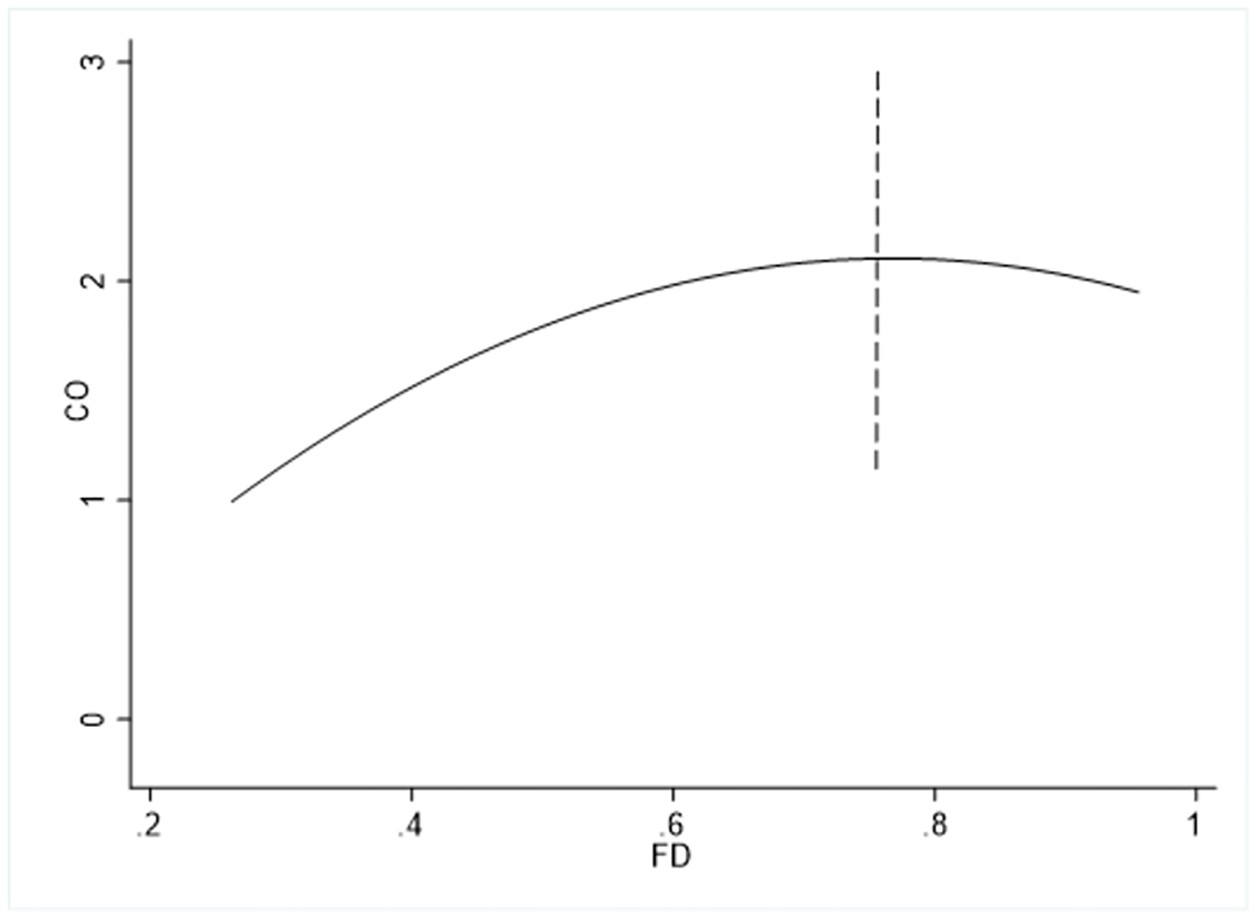
Line chart of financial development and CO2 emissions.

However, the model ignores several vital variables that may alter carbon emissions, such as foreign trade [44], urbanization level [45] and industrialization level [46]. Consequently, it is worthwhile to incorporate these variables into the STIRPAT model, extended as follows:

$$lnCO_{it}=\alpha_{0}+\alpha_{1}FD_{it}+\alpha_{2}WGI_{it}+\alpha_{3}lnRGDP_{it}+\alpha_{4}{\left(lnRGDP_{it}\right)}^{2}+\alpha_{5}lnTRA_{it}+\alpha_{6}lnPOP_{it}+\alpha_{7}lnURB_{it}+\alpha_{8}lnIND_{it}+\varphi_{it}$$ (3)

where *CO* represents per capita CO2 emissions, and we also use total carbon emissions as a surrogate variable for robustness testing. *FD* refers to the level of financial development, which includes the development of financial institutions and financial markets. *WGI* is the governance quality, including three indicators: political governance, economic governance and institutional governance. *RGDP*, *TRA*, *POP*, *URB* and *IND* represent the level of economic development, trade openness, population size, urbanization, and industrialization, respectively, and the above variables have been logarithmic to eliminate heteroscedasticity. This article also introduces the squared term of economic development into the equation to examine the applicability of the EKC. In addition, considering that the impact of financial development on CO2 emissions may have a nonlinear form, Fig 2 depicts a line graph of the relationship between financial development and CO2 emissions in G20 countries. We initially found that the effect of financial development on carbon emissions has an inverted “U” shape.

Therefore, the square term of financial development is introduced into formula (3), and the specific form is as follows:

$$lnCO_{it}=\beta_{0}+\beta_{1}FD_{it}+\beta_{2}{\left(FD_{it}\right)}^{2}+\beta_{3}WGI_{it}+\beta_{4}lnRGDP_{it}+\beta_{5}{\left(lnRGDP_{it}\right)}^{2}+\;\beta_{6}lnTRA_{it}+\beta_{7}lnPOP_{it}+\beta_{8}lnURB_{it}+\beta_{9}lnIND_{it}+\sigma_{it}$$ (4)

To investigate the comprehensive impact of governance level and financial development on reducing CO2 emissions, the interaction term of financial development and governance level is added to (3), and the equation is set as follows:

$$lnCO_{it}=\gamma_{0}+\gamma_{1}FD_{it}+\gamma_{2}FD_{it}\times WGI_{it}+\gamma_{3}WGI_{it}+\gamma_{4}lnRGDP_{it}+\gamma_{5}{\left(lnRGDP_{it}\right)}^{2}+\gamma_{6}lnTRA_{it}+\gamma_{7}lnPOP_{it}+\gamma_{8}lnURB_{it}+\gamma_{9}lnIND_{it}+\sigma_{it}$$ (5)

We pay special attention to the sign of the coefficients of FD_it_ × WEI_it_ in (5). If γ_2_ > 0, meaning that the level of governance strengthens the role of financial development in promoting carbon emissions; if γ_2_ < 0, conveying that financial development can effectively alleviate carbon emissions under the condition of good governance.

Table 2 reports the statistics of all variables and the correlations between the investigated proxies. The per capita CO2 emissions variation is between -0.223 and 3.011 metric tons per capita; the FD index varies between 0.263 and 0.956; the variation for WGI proxies is between -0.912 and 1.677 for WGI index, between 5.219 and 10.954 US$ of RGDP; trade openness varies from 2.897 and 4.659; the population size, urbanization, and industrialization range from 16.755, 3.312 and 2.844 to 21.06, 4.522 and 4.201, respectively. In addition, the RGDP series has the highest correlation with WGI, which preliminarily supports the positive role of governance quality in economic development. The correlation between economic development and per capita CO2 emissions is the second highest, demonstrating that the good performance of the economy will stimulate carbon emissions. Moreover, it can be seen that there are no outliers in the selected variables, and the correlation coefficients between the explanatory variables are all lower than 0.8, which suggests that there is no multicollinearity.

**Table 2:** Summary statistics and correlations.

	CO	FD	WGI	RGDP	TRA	POP	URB	IND
Mean	1.816	0.623	0.460	8.909	3.883	18.433	4.259	3.338
Standard deviation	0.792	0.213	0.820	1.503	0.370	1.114	0.269	0.286
Min	-0.223	0.263	-0.912	5.219	2.897	16.755	3.312	2.844
Max	3.011	0.956	1.677	10.954	4.659	21.06	4.522	4.201
CO	1							
FD	0.362	1						
WGI	0.574	0.463	1					
RGDP	0.754	0.355	0.783	1				
TRA	0.229	-0.054	0.023	0.096	1			
POP	-0.535	0.073	-0.409	-0.455	-0.347	1		
URB	0.615	0.118	0.449	0.616	-0.019	-0.712	1	
IND	-0.064	-0.046	-0.563	-0.385	0.344	0.142	-0.311	1

## 4. Results and discussion

### 4.1. Benchmark estimation

Table 3 reports the benchmark regression results for the full sample. Column (1) displays the regression results of Eq (3), and financial development has been empirically found to be positively related to CO2 emissions, and each additional unit of financial development will lead to an increase of 26.8% per capita CO2 emissions. The findings are in line with [47–49]. The main reason can be considered as the development of the financial sector stimulates CO2 emissions by promoting economic growth via savings and investment. Besides, financial development devotes carbon emissions through industrial pollution and greenhouse gases from industrialization and energy consumption as well [50]. Column (2) reports the estimation results of Eq (4), the primary term of financial development is positive at the 1% level. In contrast, the quadratic term is negative at the same significance, showing that the relationship between financial development and CO2 emission presents an inverted “U” shape. The possible reason lies in the effect of technological improvement will be exhibited when finance develops to a certain extent, and technological progress reduces the energy consumption of economic growth, or produces more alternative clean products to suppress CO2 emissions [7]. We can calculate that when the per capita CO2 emission reaches the maximum, the value of the financial development index is about 0.760, and that is to say, financial development will promote carbon emissions when the index is lower than 0.760; otherwise, financial development will play an inhibitory role. Column (3) states the estimation of Eq (5), which is the core contribution of this study, showing the nexus between governance quality and financial development in mitigating the deterioration of environmental quality. Obviously, the interaction term’s coefficient is remarkably negative, which conveys that financial development can effectively mitigate carbon dioxide emissions under good governance. This result is in line with [51, 52], who confirmed that financial development if accompanied by good governance conditions, will have a positive impact on environmental quality. Columns (4, 5) denote the effect of the interaction term of financial institution development-governance quality and financial market development-governance quality on CO2 emissions, respectively. The empirical results show that financial institution development contributes to carbon emissions while the coefficient of the interaction term is significantly negative, confirming that the development of financial institutions can substantially inhibit carbon emissions under a good governance environment. However, neither the financial market development nor the interaction term passed the 10% level, demonstrating that the development of the financial market has no obvious influence on CO2 emissions.

**Table 3:** Results of benchmark estimation.

	(1)	(2)	(3)	(4)	(5)
FD	0.268**	2.019***	0.865***		
	(0.135)	(0.439)	(0.154)		
FD^2^		-1.506***			
		(0.360)			
FD*WGI			-0.815***		
			(0.119)		
FI				0.447***	
				(0.103)	
FI*WGI				-0.755***	
				(0.0916)	
FM					0.0931
					(0.090)
FM*WGI					-0.092
					(0.087)
WGI	0.184***	0.223***	0.703***	0.670***	0.240***
	(0.052)	(0.0513)	(0.090)	(0.075)	(0.075)
RGDP	0.352***	0.304***	0.232**	0.239**	0.298**
	(0.119)	(0.117)	(0.113)	(0.110)	(0.120)
RGDP^2^	-0.0225***	-0.0191***	-0.0145**	-0.0134*	-0.0191**
	(0.007)	(0.007)	(0.007)	(0.007)	(0.008)
TRA	-0.143***	-0.130***	-0.101**	-0.0441	-0.120***
	(0.042)	(0.041)	(0.040)	(0.038)	(0.042)
POP	0.319***	0.198*	0.0727	0.228**	0.350***
	(0.106)	(0.108)	(0.107)	(0.096)	(0.106)
URB	1.901***	1.921***	2.051***	1.916***	1.956***
	(0.136)	(0.133)	(0.130)	(0.128)	(0.140)
IND	0.782***	0.728***	0.733***	0.760***	0.755***
	(0.089)	(0.088)	(0.084)	(0.082)	(0.090)
EKC	YES	YES	YES	YES	YES
Obs	399	399	399	399	399
R^2^	0.566	0.586	0.615	0.635	0.564

Note

*, ** and *** indicate the significance level at 10%, 5% and 1%, respectively; standard errors are in parentheses. All these symbols are the same for the following tables.

Additionally, as regards the governance quality, it will advance per capita CO2 emissions from the estimated results of all models, which is an unexpected result. That can be attributed to the following two aspects: On one hand, among the G20 countries, only a few developed countries have achieved carbon peaks through natural processes or driven by environmental policies, while most other nations have not yet, and their carbon emissions are closely related to economic development. On the other hand, most emerging and developing countries have not formed a sound institutional guarantee for environmental governance, and the focus of their arrangements may deviate from environmental protection, but instead lies in the political environment and economic performance. Looking at the results of other control variables, we find that the coefficient of per capita GDP is significantly positive, while the coefficient of its square term is negative, meaning that the nexus between economic growth and CO2 emissions presents an inverted “U”-shaped trend. The results of this paper confirm the existence of the EKC hypothesis for CO2 emissions [53, 54]. Trade liberalization has a considerable inhibitory effect on CO2 emissions, which is consistent with [55], revealing that these countries adopt relatively strict environmental regulations, and international trade brings the flow of green technologies and commodities. At the same time, the “polluter paradise hypothesis” is not supported in the overall scope of G20 nations. Population size, urbanization and industrialization levels are significantly positively correlated with carbon emissions, which are in line with [28, 56], showing that these three factors will aggravate local environmental degradation and hinder sustainable economic development.

### 4.2. Robustness checks

In order to test the reliability of the benchmark estimation results, this article uses three different methods for robust testing. First, considering that the impact of explanatory variables on CO2 emissions may have a certain lag, the explanatory variables were lagged by one period and then regressed to verify the robustness of the benchmark estimation [57]. The results are given in Table 4 columns (1, 2). Second, in the case of different measurement methods, the explained variables will affect the results; hence the explained variables are represented by the total amount of CO2 emissions to analyse their robustness [58]. The results are displayed in columns (3, 4). Third, to avoid the impact of short-term economic cycle fluctuations, the three-year average of each variable is taken to construct a new variable, and finally five-period sample data can be obtained [59]. Columns (5, 6) are the estimated results.

**Table 4:** Robustness checks.

	Lag by one stage		Total CO2 emissions		Sample staging	
	(1)	(2)	(3)	(4)	(5)	(6)
FD	1.698***	0.728***	1.996***	0.846***	2.370***	1.121***
	(0.440)	(0.155)	(0.435)	(0.153)	(0.890)	(0.309)
FD^2^	-1.279***		-1.495***		-1.770**	
	(0.365)		(0.357)		(0.736)	
WGI	0.206***	0.620***	0.227***	0.700***	0.246**	0.869***
	(0.0517)	(0.0907)	(0.0509)	(0.0894)	(0.101)	(0.183)
FD*WGI		-0.711***		-0.803***		-1.015***
		(0.121)		(0.118)		(0.235)
Control	YES	YES	YES	YES	YES	YES
EKC	YES	YES	YES	YES	NO	NO
Obs	380	380	399	399	133	133
R^2^	0.579	0.603	0.729	0.747	0.615	0.655

Compared with the columns (2, 3) of Table 4, the results of the above three different methods show that the coefficients of financial development, its square term, and the interaction term with governance quality have changed in magnitude, whilst their signs and significance are consistent with the previous results, which fully proves that the benchmark estimation in this paper has strong robustness.

### 4.3. Further analysis

#### 4.3.1. The heterogeneity test of economic development

The extent to which financial development affects CO2 emissions may vary country-by-country. Thus we divided the sample into two groups of developing and developed countries for regression to test the heterogeneity sourced from different types of countries [47]. Developing countries include Argentina, Brazil, China, India, Indonesia, Turkey, Saudi Arabia, South Africa, Russia and Mexico; developed countries include Australia, Canada, France, Germany, Japan, South Korea, Italy, the United States and the United Kingdom. The first and last four columns of Table 5 report sample regressions for developing and developed countries, respectively.

**Table 5:** Heterogeneity test results of economic development level.

	Developing countries				Developed countries			
	(1)	(2)	(3)	(4)	(5)	(6)	(7)	(8)
FD	1.863***	-0.119			-2.418***	1.682***		
	(0.596)	(0.159)			(0.470)	(0.397)		
FD^2^	-1.653***				1.835***			
	(0.544)				(0.354)			
FI			0.0207				0.762	
			(0.136)				(0.483)	
FM				-0.413***				1.325***
				(0.094)				(0.262)
WGI	0.073	0.766***	0.543***	0.458***	0.394***	1.281***	1.021***	0.989***
	(0.052)	(0.113)	(0.100)	(0.0997)	(0.0672)	(0.181)	(0.226)	(0.130)
FD*WGI		-1.259***				-1.193***		
		(0.186)				(0.261)		
FI*WGI			-0.810***				-0.827**	
			(0.165)				(0.324)	
FM*WGI				-0.779***				-0.826***
				(0.170)				(0.181)
Control variable	YES	YES	YES	YES	YES	YES	YES	YES
EKC	NO	NO	NO	NO	NO	NO	YES	YES
Obs	210	210	210	210	189	189	189	189
R^2^	0.852	0.852	0.850	0.839	0.734	0.725	0.726	0.732

The results show that the financial development coefficient of developing countries is significantly positive, and its quadratic coefficient is significantly negative, demonstrating that the link between financial development and CO2 emissions presents an inverted “U” shape, while it is a “U”-shaped trend in developed countries. The result in developing countries is consistent with [60], and they proposed that developing nations should balance the relationship between financial development and environmental quality. Indeed, it applies to developed countries as well in the paper. Based on the stated above, the role of financial development on CO2 emissions is indeed heterogeneous among countries with different economic levels. Therefore, developed countries are recommended to moderately slow down financial development to reach the inflextion point. For developing countries, it is necessary to vigorously promote financial development to a certain extent with the purpose of carbon emissions reduction. Then compare the interaction coefficients of financial development, institution development and market development with the governance quality of these two groups of countries, respectively. We can find that the coefficients of these three interaction terms are all significantly negative in both developing and developed countries, identifying that the governance level and financial development show an alternative relationship in the CO2 emission reduction. It can be concluded that good governance quality can effectively mitigate the negative effect of financial development on CO2 emissions.

#### 4.3.2. Governance quality sub-indicator

Financial development includes institutions and market development. Governance quality indicators are divided into three dimensions: political governance, economic governance, and institutional governance. Table 6 reports the empirical results of the interaction between the two sub-indicators of financial development and the three dimensions of governance quality. Columns (1–3) show the estimated results of the interaction terms between institutional development and political, economic, and institutional governance, respectively, and columns (4–6) display the results of the interaction terms between market development and the above three variables.

**Table 6:** Results of governance quality sub-indicator test.

	(1)	(2)	(3)	(4)	(5)	(6)
FI	0.280***	0.490***	0.443***			
	(0.104)	(0.110)	(0.104)			
FM				0.116	0.0535	0.0700
				(0.086)	(0.096)	(0.090)
POL	0.513***			0.153**		
	(0.070)			(0.065)		
ECO		0.554***			0.155**	
		(0.067)			(0.067)	
INS			0.554***			0.195***
			(0.065)			(0.066)
FI* POL	-0.676***					
	(0.090)					
FI*ECO		-0.664***				
		(0.094)				
FI* INS			-0.642***			
			(0.082)			
FM* POL				-0.170**		
				(0.084)		
FM* ECO					0.0172	
					(0.087)	
FM* INS						-0.0583
						(0.076)
Control variable	YES	YES	YES	YES	YES	YES
EKC	YES	YES	YES	YES	YES	YES
Obs	399	399	399	399	399	399
R^2^	0.613	0.621	0.629	0.555	0.567	0.562

It can be seen that financial institutional development substantially stimulates CO2 emissions; although the sign of the coefficient of financial market development is positive, it does not pass the 10% level, which is consistent with the previous benchmark results. The growth of the financial sector is an important driver of carbon emissions [61]. Political, economic and institutional governance all have significant positive effects on CO2 emissions, which demonstrate that the governance quality indicators we selected do not have the effect of improving environmental quality in the analysis of G20 countries. We focus on the coefficients of each interaction item and find that the coefficients between financial institutional development and the three governance indicators are all significantly negative, suggesting that governance quality and the financial institution sector complement each other to reduce CO2 emissions. The coefficient of the interaction term of financial market development only with political governance was significantly negative, whilst neither with economic nor institutional governance passed the 10% level. Good political arrangements can stabilize the development of financial markets and promote the rise of green finance [62]. Therefore, the necessary measures should be taken to establish good governance quality and strengthen financial development in response to the improvement of environmental quality.

## 5. Conclusions and policy recommendations

This paper focuses on analysing the relationship between financial development and carbon emissions in G20 countries under good governance for the period 1999–2019. The main conclusions are as follows: First, financial development has a significant positive effect on CO2 emissions; when the quadratic term of financial development is introduced, the impact presents an inverted “U”-shaped trend. Second, improvements in governance quality increase CO2 emissions, which is an unexpected result. However, good governance mitigates the negative impact of financial development on environmental quality. Third, the development of financial institutions significantly increases per capita CO2 emissions, but in the context of good governance, carbon emissions can be suppressed, and financial market development does not have an obvious impact on carbon emissions. Fourth, there is an inverted “U”-shaped relationship between financial development and CO2 emissions in developing countries, while a “U”-shaped relationship in developed countries. And the good governance of these two groups of countries can effectively mitigate the negative effect of financial development on the environment.

Based on the above analysis, the following policy recommendations are put forward: First of all, although the implementation of financial development strategies in various countries serves the policy agenda and helps achieve national economic growth, in the long run, these practices are unsustainable. Thus, policymakers should shift their agenda to environmentally responsible financial practices for financial development. Then, policymakers can effectively improve environmental quality by optimizing the quality of governance. Good governance will make an important contribution to the financial sector in curbing carbon emissions via strengthening legal and institutional systems, implementing standards, and establishing effective regulators. Moreover, different countries should distinguish and clarify their respective emission reduction tasks. The financial development level and energy structure of developing countries are comparatively backward compared with developed countries. Hence, they should actively adjust the financial structure so that the financial sector can fully play its role in devoting to environmental quality. Finally, the G20 should make concerted efforts to strengthen international cooperation and formulate corresponding measures, which will help countries work together to achieve low-carbon and win-win targets.

Although this paper makes some marginal contributions to the existing literature, it still has certain limitations. Firstly, this paper uses per capita CO2 emissions to measure environmental pollution. However, other types of gases can affect the environment, such as sulfur dioxide and methane. Future research will comprehensively consider using various types of polluting gas-related data to analyse the relationship between governance quality, financial development and environmental pollution. Secondly, though our sample covers the world’s major carbon-emitting economies, including the most developed and important developing countries, it does not include enough poor economies, which is not conducive to achieving global carbon emission reduction goals and a more detailed division of reduction responsibilities. Further research should be extended to various countries around the world. Thirdly, the current study uses panel data and should be accompanied by time series estimation, focusing on individual countries for comparative analysis, such as China, the United States, Japan and Germany. This may lead to a deeper understanding of the role of governance quality and make targeted recommendations for policymakers in various countries on the reduction of carbon emissions.

## Supporting information

S1 Data (XLSX)
